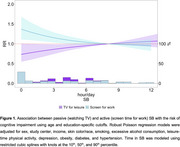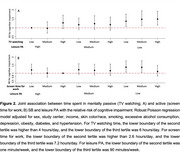# Distinct sedentary behaviors and cognitive decline: Findings of the ELSA‐Brasil study

**DOI:** 10.1002/alz70860_103889

**Published:** 2025-12-23

**Authors:** Natan Feter, Bruce Duncan, David A Raichlen, Maria Inês Schmidt

**Affiliations:** ^1^ Universidade Federal do Rio Grande do Sul, Porto Alegre, Brazil; ^2^ University of Southern California, Los Angeles, CA, USA; ^3^ Postgraduate Program in Epidemiology, Universidade Federal do Rio Grande do Sul, Porto Alegre, Rio Grande do Sul, Brazil; ^4^ Medical School, Universidade Federal do Rio Grande do Sul, Porto Alegre, Rio Grande do Sul, Brazil

## Abstract

**Background:**

Sedentary behavior (SB) has been associated with adverse cognitive outcomes. Mentally active sedentary behavior and PA may mitigate the negative impact of SB on brain health. We aimed to investigate the association between context‐specific SB, PA, and cognitive decline in middle‐aged and older adults over a 4‐year follow‐up.

**Method:**

We analyzed data from the *Estudo Longitudinal de Saude do Adulto* (ELSA‐Brasil) study. Participants were enrolled between 2012 and 2014, with a follow‐up visit between 2017 and 2019. We used the International Physical Activity Questionnaire – long form to assess leisure PA. We evaluated screen time at work or study and at leisure to categorize SB as mentally active or passive. We evaluated global and domain‐specific (memory, verbal fluency, and executive function) cognitive function at baseline and follow‐up. Incident cognitive impairment was defined using age and education‐specific cutoffs.

**Result:**

Participants (*N* = 5,795) had a mean age of 62.4 (SD: 5.8) years and were primarily female (57%). During a mean follow‐up of 4.4 (SD: 0.5) years, greater leisure screen time was associated with a higher risk of cognitive impairment, while more screen time for work or study was linked to a lower risk. Screen time for work was associated with a slower decline in global cognition (β=0.03; 95%CI: 0.01, 0.04), memory (β=0.05; 95%CI: 0.00, 0.10), language (β=0.07; 95%CI: 0.02, 0.11), and executive function (β=0.17; 95%CI: 0.11, 0.22). Leisure screen time was associated with more rapid memory decline (β=‐0.07; 95%CI: ‐0.11, ‐0.02). Medium (4‐6 hours/day: RR: 0.99; 95%CI: 0.72‐1.36) and high (≥7 hours/day; RR: 1.25; 95%CI: 0.93, 1.67) leisure screen time was not associated with higher risk of cognitive impairment in those with high PA levels (Figure 1). In individuals with high screen time for work (≥7 hours/day), low PA was not linked to a higher risk of cognitive impairment compared to those with high PA (RR≥1.29; *p* <0.05 for all categories) (Figure 2). There was no interaction between SB and PA (*p* ≥0.903).

**Conclusion:**

The association between SB and cognitive decline is context dependent. PA appears to mitigate the negative association between mentally passive SB and cognitive decline.